# Illuminating membrane structural dynamics of fusion and endocytosis with advanced light imaging techniques

**DOI:** 10.1042/BST20210263

**Published:** 2022-08-12

**Authors:** Chung Yu Chan, Youssef Faragalla, Ling-Gang Wu

**Affiliations:** 1 National Institute of Neurological Disorders and Stroke, 35 Convent Dr., Bethesda, MD 20892, U.S.A.; 2 Neurosciences Graduate Program, Stanford University, Stanford, CA 94305, U.S.A.

**Keywords:** endocytosis, exocytosis, membrane fission, membrane fusion, super-resolution imaging, synaptic transmission

## Abstract

Visualization of cellular dynamics using fluorescent light microscopy has become a reliable and indispensable source of experimental evidence for biological studies. Over the past two decades, the development of super-resolution microscopy platforms coupled with innovations in protein and molecule labeling led to significant biological findings that were previously unobservable due to the barrier of the diffraction limit. As a result, the ability to image the dynamics of cellular processes is vastly enhanced. These imaging tools are extremely useful in cellular physiology for the study of vesicle fusion and endocytosis. In this review, we will explore the power of stimulated emission depletion (STED) and confocal microscopy in combination with various labeling techniques in real-time observation of the membrane transformation of fusion and endocytosis, as well as their underlying mechanisms. We will review how STED and confocal imaging are used to reveal fusion and endocytic membrane transformation processes in live cells, including hemi-fusion; hemi-fission; hemi-to-full fusion; fusion pore opening, expansion, constriction and closure; shrinking or enlargement of the Ω-shape membrane structure after vesicle fusion; sequential compound fusion; and the sequential endocytic membrane transformation from flat- to O-shape via the intermediate Λ- and Ω-shape transition. We will also discuss how the recent development of imaging techniques would impact future studies in the field.

## Introduction

**Figure 1.**
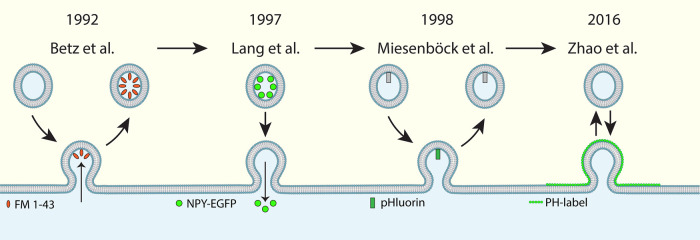
Timeline of imaging probes in the study of membrane fusion and fission. Left: Schematic illustrating the mechanism of FM 1–43 uptake in studying neurotransmitter release and endocytosis from Betz et al. [1], where the label attaches to the Ω-profile following release and stays in the vesicle following endocytosis. Center left: Schematic illustrating the mechanism of NPY-EGFP release in studying vesicle release by Lang et al. [2], where NPY-EGFP is expressed and disappears the following release. Center right: Schematic illustrating the mechanism of the pHluorin-tag by Miesenböck et al. [4] where the pHluorin signal is dampened by the low pH environment of the vesicle following fission and prior to fusion, but increases following fusion pore opening due to the higher pH environment of most physiological solutions. Right: Schematic illustrating the mechanism of the PH label in Zhao et al. [7], where the PH label surrounds the inner leaflet of the Ω-profile following fusion and during hemifusion, and disappears from the vesicle following fission or full-collapse fusion.

In the study of fundamental cell processes, the advancement of microscopy and imaging tools has accelerated our understanding in an innumerable way over the past half-century. The study of dynamic processes such as membrane fusion and endocytosis is an example of this progress. Early studies have successfully detected vesicle exo- and endocytosis using fluorescent FM1–43 to label exocytic and endocytic vesicles (Figure 1) [1], which represented the earliest live-cell imaging of exo- and endocytosis captured with epi-fluorescence microscopy [1,5]. However, these early imaging techniques were unable to visualize the membrane transformation underlying vesicle exocytosis and endocytosis. The revolution of fluorescent protein labeling brought many new interesting applications to study proteins and lipids involved in membrane fusion and fission, including phosphatidylinositol-4,5-bisphosphate (PIP2)-binding lipases and neuropeptides in vesicles [2–4,6]. The labeling of the PHδ-phospholipase C domain with a fluorescent protein allowed for the visualization of PIP2 which binds to the aforementioned domain [3]. The labeling of this domain (referred to as PH from this point on for brevity) does not just provide a label for a lipid that is heavily involved in exocytosis and endocytosis [2,3], but also provides a label of the cytoplasmic leaflet of the plasma membrane (PM); allowing for direct visualization of membrane fusion and fission events that can facilitate characterizations of vesicle and endocytic structure size, shape, and quantity with ease (Figure 1) [7,8].

The fluorescent labeling of vesicle cargo such as neuropeptide Y (NPY) and fluorescent false neurotransmitter (FFN) also allowed for the measurement of vesicle fusion events (Figure 1) [2,9]. FFN in particular is an attractive tool to study release as it does not require transfection or transgenic manipulation. It is a monoamine analog that is taken up by the vesicular monoamine transporter (VMAT) after incubation of the label in cells over a range of minutes; after which point the cells are washed and vesicles are labeled [10]. In addition, the creation of the pH-sensitive pHluorin-tag allowed for characterization of fusion pore opening (increase in pH value and pHluorin signal) and closure (decrease in pH value and pHluorin signal) that is still actively used in synaptic vesicle release studies to this day (Figure 1) [4].

In conjunction with new microscopy methods such as total internal reflection microscopy and two-photon microscopy that can record time series data on the order of milliseconds, the field was on the cusp of groundbreaking findings in the dynamics of membrane fusion and fission [11,12]. Takahashi et al. [12] used two-photon microscopy to image insulin fusion pore dynamics in live intact mouse pancreatic islets, being able to discriminate nanoscale pore sizes using different extracellular dyes, obtaining the first observation of fusion pore dynamics at a high spatiotemporal resolution in living tissues. The development of evanescent-wave and total internal reflection microscopy in studying membrane fusion and fission events also aided the field, allowing for high-temporal resolution imaging of these phenomena at the PM level as described in previous work by Oheim et al. [13]. The first studies of membrane fusion and fission using super-resolution microscopy, particularly stimulated emission depletion (STED) microscopy, focused on the antibody labeling of synaptotagmin and its uptake by cells as a marker of vesicle fusion [9,14]. Despite this advancement in imaging probes and in microscopy, the three-dimensional nature of membrane fusion and fission dynamics means that it is difficult to study these phenomena on the base of the PM alone. The fact that many of these membrane fusion or fission phenomena are occurring at a spatial resolution below the diffraction limit also means it is difficult to separate structures that are either directly on membrane structures or simply surrounding them. Therefore, imaging techniques combining the power of state-of-the-art microscopy methods with the imaging tools developed to study membrane dynamics are necessary to continue the study of membrane fusion and fission.

## Vesicle fusion and exocytosis

Vesicle fusion has been imaged by many techniques, including total internal reflection fluorescence (TIRF) microscopy [15] or polarized TIRF imaging [11], two-photon imaging with extracellular dye [12], and interference reflection microscopy [16]. The earliest study of single vesicle using confocal imaging had shown the potential of imaging the dynamic events at an appropriate temporal and spatial resolution [17]. However, there was no in-depth study of the post-fusion vesicle structures using the available methods. In the following sections, recent findings that have filled this knowledge gap by advanced imaging techniques will be highlighted.

### Observation of fusion-generated Ω-profiles

**Figure 2.**
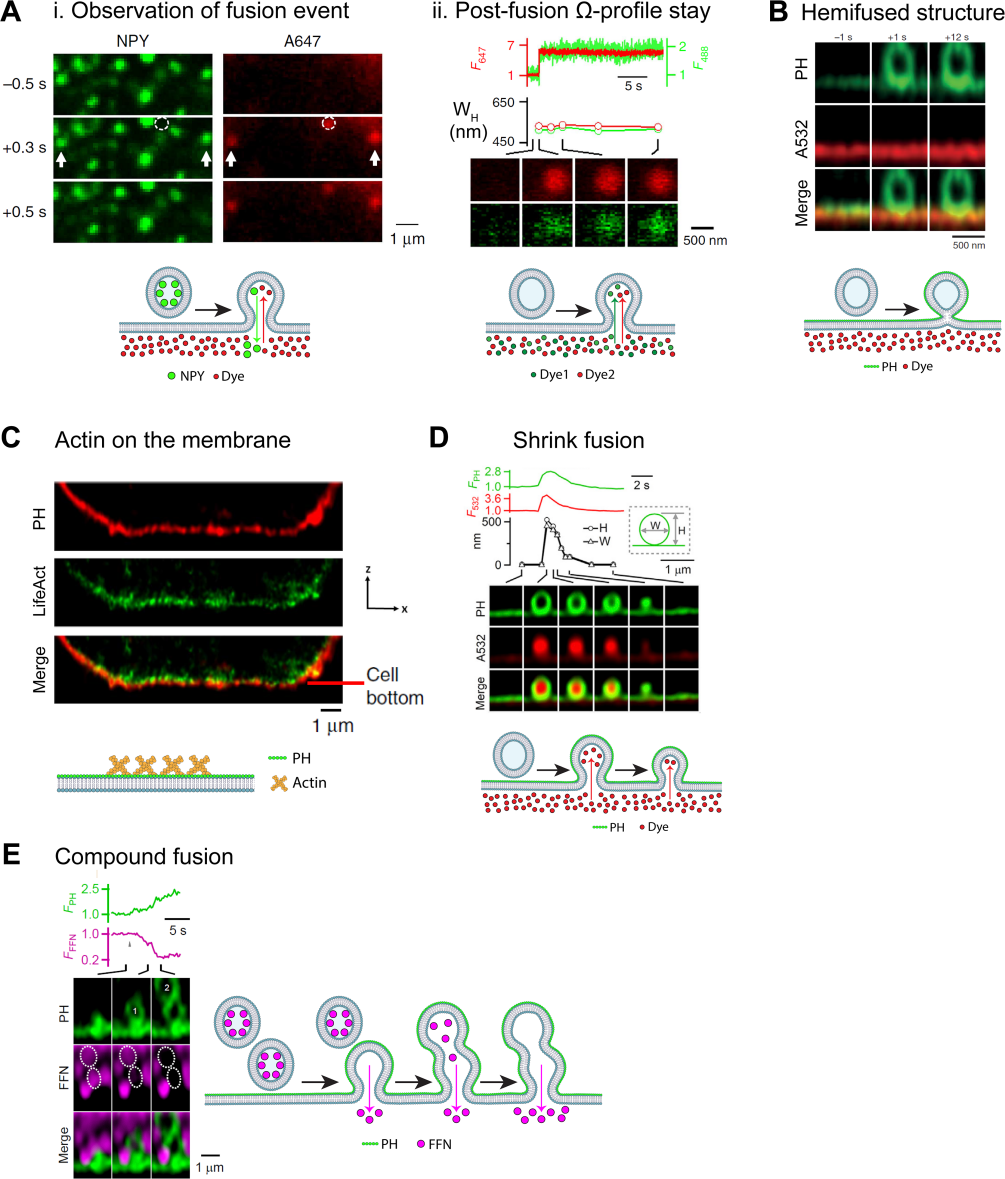
Highlights of the recent understanding of vesicle fusion with advanced light imaging techniques. (**A**)(i) Two-color confocal imaging of NPY-EGFP (green) and A647 (red) for a fusion event during different time point of stimulation (−0.5 s to +0.5 s) [18]. Arrows show NPY-EGFP content release together with the appearance of A647 spots. The schematics shows that after a vesicle is fused, NPY are released while A647 diffuse into the fused vesicle. (**A**)(ii) Upper panel: Further analysis of two dyes imaging (Red: A647 and Green: A488) of a single spot showing the Ω-profile staying post-fusion at a time interval of 15 ms. (F_647_: Fluorescence of A647; F_488_: Fluorescence of A488). Lower panel: The width (*W*_H_) of the spot remains constant across the time measured [18]. (**B**) Two-color STED XZ images of PH-EGFP and A532 for a PH only Ω-profile after stimulation, indicating a hemi-fused structure [7]. Schematics shows that the hemi-fused structure remains impermeable to extracellular dye. (**C**) Two-color STED XZ images of PH–mPapaya and Lifeact-TagGFP2 showing the visualization of actin with the membrane [19]. (**D**) Two-color STED XZ images of PH-mNeonGreen and A532 showing the dynamics of shrink fusion [20]. Upper panel: Analysis of a single spot (Green: PH and Red: A532) showing the Ω-profile shrinking across the time (F_PH_: Fluorescence of PH; F_532_: Fluorescence of A532). Lower panel: The width and height of Ω-profile are analyzed, showing the diminishing width and height of a shrinking Ω-profile (as depicted in the schematics below). (**E**) Two-color STED XZ images of PH-mNeonGreen and FFN511showing release of FFN511 during sequential compound fusion [21]. As shown in the schematics below, after one vesicle is fused and releases the FFN, a second vesicle is then fused on the existing vesicle to release its content of FFN.

The use of extracellular dyes by Chiang et al. [18] would expand the scope by distinguishing different modes of post-fusion events. In this detailed study, the fusion-generated Ω-profile — indicative of vesicle fusion with the PM-was first imaged with simultaneous confocal imaging of neurotransmitter marker NPY-EGFP and the extracellular dye Alexa Fluor 647 (A647) following patch clamp-stimulated vesicle release of the adrenal chromaffin cell model. Although NPY-EGFP had labeled instances of vesicle release in prior studies [2], the addition of A647 allowed for the visualization of the post-fusion structures previously unseen at a high spatiotemporal resolution in a live-cell preparation. Single spots representing individual NPY-EGFP release were shown to coincide with A647 appearance (Figure 2A), indicating the formation of the stable Ω-profile. These new methods, with the use of confocal microscopy as well as vesicular lumen and extracellular labels, have shown the dynamics of post-fusion structure formation and provided support for a model that merges the phenomena of exocytosis and endocytosis with the structure of the Ω-profile as the centerpiece. The methods have provided a platform for others to build upon, particularly in the *in vitro* studies of vesicle fusion [22].

### Observation of hemi-fused structure

To gain deeper knowledge of the fusion process, one should study the possible intermediate structures. It has long been proposed that vesicle fusion involve an intermediate hemi-fused structure, where the outer leaflet of the vesicle membrane fuses with the inner leaflet of the PM while the inner leaflet of the vesicle membrane remains closed without any content release. Another competing fusion hypothesis involving protein-lined pore formation was suggested in the past [23–28], but not until recently the spatiotemporal resolution was sufficient to confirm the presence of these structures. By using confocal and super-resolution STED microcopy, Zhao et al. [7] reported the visualization of a hemi-fused Ω-profile in live neuroendocrine chromaffin cells and pancreatic β-cells for the first time. This study employed a similar approach as Chiang et al. [18] with the labeling of membrane (predominantly PH at the cytosolic leaflet) and extracellular dyes in conjunction with the aforementioned imaging and stimulation protocol. Hemi-fused structure was observed in confocal in XY-plane and was better visualized in the STED XZ-plane images due to its significantly superior spatial resolution (Figure 2B). Another study also demonstrated the visualization of soluble *N*-ethylmaleimide-sensitive factor (NSF) attachment protein receptor (SNARE)-mediated hemifusion between giant unilamellar vesicles by confocal imaging and fluorescence recovery after photobleaching (FRAP) [29]. These works show the promise of combining imaging and microscopy tools to study previously unseen phenomena such as hemi-fission and hemi-fusion; and provides the foundation and techniques for the further study in live cells.

### Actin involvement in providing tension during vesicle fusion

Cytoskeletal filamentous actin has long been considered a molecule that may regulate exocytosis [30,31]. Recent studies shed new light on many crucial roles of actin [32], such as facilitation of vesicle movement to the readily releasable pool (RRP), and involvement in shrink fusion [18,33,34]. By employing confocal and super-resolution STED imaging, recent studies showed that fused vesicle shrinking is due to a squeezing force provided by the osmotic pressure difference between the intracellular and the extracellular solution, whereas actin provides tension at the PM to reel off the fusing vesicle membrane which is of lower tension due to the squeeze by the osmotic pressure [19,20]. These studies are consistent with an early observation that actin and myosin II may speed up the content release of chromaffin granules, which led to a proposal that actin and myosin II may squeeze granules to speed up release [35]. It also built on the method of not just labeling lipids but proteins as well from Zhao et al. [7] (where the SNARE protein vesicle-associated membrane protein 2 (VAMP2) was imaged), and the addition of the actin tag Lifeact (Figure 2C) allowed for the investigation of proteins in mediating membrane fusion at a high spatiotemporal resolution [19].

### Imaging the dynamics of vesicle content release modes

Based on the findings in Ω-profile shrinking [18,19], Shin et al. extended the study into the mechanism of fusion modes of exocytosis. Using similar techniques to previous studies, the authors found that instead of full collapse, the extent of content release was dominated by shrink fusion, where the Ω-profile shrank but the fusion pore did not change in diameter, and profile size is maintained by actin counteracting inward-facing osmotic pressure forces [20]. Another substantial finding is that instead of kiss-and-run, enlarge fusion — in which Ω-profiles grow while maintaining a narrow pore — reduces content release [20]. These results corroborate with an earlier study showing that the membrane capacitance up-step that may reflect single vesicle fusion can be followed by a larger down-step in mast cells containing extremely large vesicles [36], suggesting that enlarge fusion may not be limited to chromaffin cells. While previous studies have focused on vesicle release at the PM as the primary site of release, the concept of sequential compound fusion has originally been implicated in studies of eosinophil degranulation using capacitance measurements [37] and also fluorescence imaging with 40 ms time resolution [38]. By using fast STED imaging and previously described membrane, extracellular space, and vesicle labels, Ge et al. [21] visualized sequential compound fusion where the vesicles fuse with previously formed and large Ω-profiles in excitable cells (Figure 2E). While the concept of compound fusion was originated from previous work [37–39], this is the first time it has been observed and visualized in sufficient spatiotemporal resolution, and provide new theories for how docking, priming, and other events preceding fusion could be facilitated by these preformed Ω-profiles [21] (Figure 2E).

### Understanding the fusion pore dynamics

**Figure 3.**
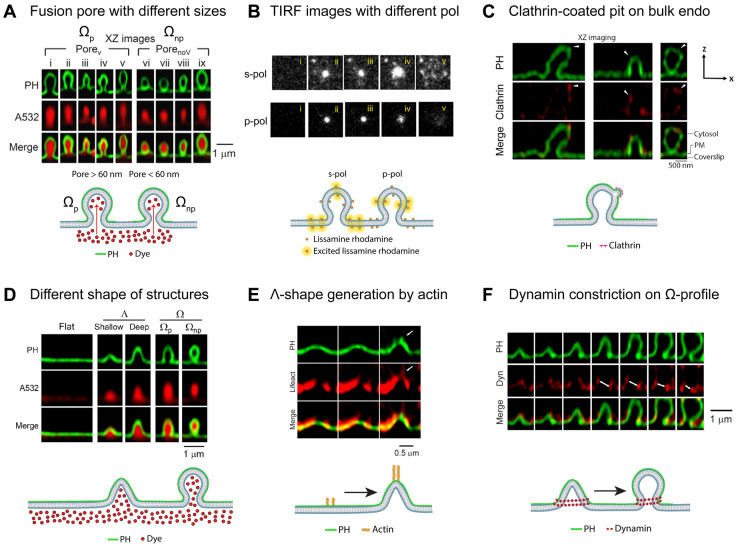
Summary of the recent understanding of fusion pore and vesicle endocytosis with advanced light imaging techniques. (**A**) Two-color STED images (PH and A532) of sampled Ω-profiles with fusion pores visualized (Ω_p_) and not visualized (Ω_np_) because of the spatial resolution limit of 60 nm on that STED equipment [50]. Schematics below show that dye can freely diffuse into both Ω_p_ and Ω_np_. (**B**) TIRF images showing total fluorescence intensity profiles of a fusion event recorded with s-pol (upper panel) or p-pol excitation (lower panel) [51]. The fluorophore lissamine rhodamine with an excitation dipole parallel to the membrane will be excited more efficiently in the supported bilayer (SBL) than the small unilamellar vesicles (SUV) using s-pol excitation. Schematics below show the orientation of lissamine rhodamine that can be excited in s-pol or p-pol configurations. (**C**) Two-color STED XZ images of PH-mNeonGreen (Green) and Clathrin-mTFP1 (Red) showing three large Ω-profiles associated with clathrin-mTFP1 puncta (white triangles designate membrane protrusions as potential clathrin-coated pits) [52]. (**D**) Examples of Flat, Λ (shallow or deep), and Ω (Ω_p_ or Ω_np_) in cells by using two-color STED imaging [8]. (**E**) STED XZ images of PH-mNeonGreen and lifeact-mTFP1 showing actin filament recruitment, attachment at, and movement with the growing Λ’s tip [53]. White arrow indicates spike-like protrusion of the growing actin filaments. Schematics below demonstrate the formation of Λ from flat membrane with the help of actin. (**F**) Two-color STED XZ images of PH-mNeonGreen and dynamin 1-mTFP1 puncta surrounds and move with constricting Λ‘s base and constricting Ω’s pore [53]. Schematics below depict the formation of Ω-profile from Λ-profile with the constriction of dynamin.

Membrane pores are found across multiple cell contexts [40–46]. Early detailed studies of fusion pore dynamics were carried out in chromaffin cells by patch-clamp capacitance measurements, where the relation between fusion pore conductance dynamics and transmitter release has been characterized [47,48]. The conductance recording method may reveal initial fusion pores, but difficult to resolve pores larger than ∼5 nm [49]. With super-resolution STED microscopy, Shin et al. [50] visualized dynamics of fusion pores with size up to 490 nm (Figure 3A) controlled by actin forces mediating pore opening and dynamin forces mediating constriction as they play complementary roles to regulate pore size. Using TIRF, Guček et al. [54] showed that fusion pore expansion is governed by cyclic AMP-sensor Epac2 to recruit amisyn and dynamin-1 to the pore in insulin-secreting beta-cells. Customized polarization-controlled TIRF microscope was built and employed to measure content releases from the pore of proteoliposomes with single molecule sensitivity and ∼15 ms temporal resolution (Figure 3B) [51]. Regulation of fusion pore by vesicle cholesterol was also demonstrated in lactotroph with structured illumination microscopy [55]. The understanding of fusion pore dynamics has been greatly enhanced by advanced light imaging techniques.

Due to the limit of spatial and temporal resolution, current imaging techniques are difficult to resolve the initial fusion pore within ∼5 nm at millisecond resolution [50], whereas cell-attached capacitance recordings are capable of estimating such a small and fast fusion pore dynamics [40,49]. This raises a possibility of combining cell-attached capacitance recording with STED imaging to resolve the entire fusion pore dynamics from less than 5 nm up to hundreds of nanometers. Alternatively, the development of imaging techniques with much higher spatial and temporal resolution is needed to solve this problem.

## Vesicle fission and endocytosis

Early studies on imaging endocytic pathway rely on lipophilic dyes such as FM dyes, which fluoresce strongly when bound to the membrane [56], can be taken up via endocytosis (staining). Since then, many light imaging platforms have been used to study endocytosis, such as TIRF [57], automated super-resolution imaging [58], and simultaneous two-wavelength axial ratiometry (STAR) microscopy [59]. Latest studies have provided insights of new models in vesicle endocytic dynamics.

### Clathrin-mediated endocytosis cooperation with bulk endocytosis

In the nervous and endocrine systems, intense stimulation rapidly deplete exocytotic content-filled vesicles from the cells, and that mechanisms to retrieve fusing vesicles in endocytosis need to occur in milliseconds to tens of seconds [40,60]. Of all the known endocytic modes, clathrin-mediated endocytosis (CME) is the most prominent one because the generation of clathrin-coated patches, pits, and vesicles (∼30–100 nm) can be observed from the PM by various techniques [61–63]. In general, CME is thought to be generated from flat PM regions [43,60,64–66] but a study several decades ago also proposed that clathrin-coated vesicles can also be generated from preformed PM invaginations [67]. In this pioneering study, however, the model was based on only a few examples without robust statistics. To explore the alternate origin of CME in a recent study, Arpino et al. [52] employed super-resolution STED microscopy together with other techniques to show that large Ω-shaped or dome-shaped PM invaginations were primary sites for clathrin-coated pit generation in neuroendocrine chromaffin cells after stimulation (Figure 3C). Those sites were previously thought of as the precursor of bulk endocytosis and clathrin-coated pits were more densely packed at invaginations rather than flat membranes. The results suggested that CME closely collaborates with bulk endocytosis to enhance endocytic capacity in active secretory cells. This again demonstrates the ability of advanced light imaging techniques to provide evidence to support the previous study in the field.

### Flat-to-round membrane transformation and its regulation

Excitable cells like neurons and endocrine cells develop multiple endocytic mode to cope with highly dynamic demands in physiological conditions. Shin et al. [8] discovered that flat membrane is transformed into different shaped vesicles via invagination (Figure 3D). Unexpectedly, most endocytic vesicle formation is not directly from flat-membrane-to-round-vesicle transformation as generally accepted, but through preformed profiles (structures or invaginations formed before stimulation) undergoing endocytic modular transitions. Decades of studies also suggest that the membrane transforming force may generate in part from cage-like structures coating the endocytic vesicle and formed by multimerization of clathrin or other vesicle coat-proteins [62,63,68]. However, in the absence of core coat-proteins such as those in clathrin-independent endocytosis (CIE) [69], what type of physical forces mediate non-coated-membrane transformation remain largely unclear. Shin et al. [53] visualized how this process was regulated: actin and dynamin generated a pulling force transforming flat membrane into Λ-shape (Figure 3E); subsequently, dynamin helices surround and constrict the base to Ω-profile, and then constrict to O-shaped vesicles (Figure 3F). The novel mechanical roles of actin and dynamin reported here may also shed light on those involved in cell migration, cell fusion, cell division, neuronal branching, and cell-shape formation [68,70,71]. These new imaging data challenge the current view that membrane flat-to-round transformation mediates all diverse endocytic modes, which may apply broadly to the endocrine and nervous system, and many other systems using various modes of endocytosis.

## Conclusion and future outlook

These new techniques have revolutionized our understanding of the variety of dynamics underlying vesicle fusion and endocytosis, that until recently were previously unobservable either in dimensions of space or time. The field now has a greater understanding of the dynamics underlying changes in vesicle size, pore size, fusion states, compound fusion, and CME over time thanks to the advent of super-resolution microscopy. With new probes and labels, the roles of previously speculated proteins such as actin and dynamin among others can now be confirmed and their roles in mediating membrane fusion and fission can be now observed over larger periods of time.

As microscopy methods improve in spatial resolution, so will the understanding of membrane fusion and fission. The promise of minimal photon fluxes (MINFLUX) microscopy, touted to image cellular contents at <10 nm spatial resolution [72,73], has already demonstrated its capability in recent studies [74–76]. New labeling techniques such as nanobodies and click chemistry have been developed to obviate the bulky size of antibodies and fluorescent protein/tag [77–80]. This will give greater insight into the fusion and fission pore sites. Fast three-dimensional imaging of these phenomena will also improve the dynamics of these three-dimensional structures, particularly with the investigation of actin and cytoskeletal forces using methods such as grazing incidence structured illumination microscopy and lattice light sheet microscopy [81,82]. The ability to also increase the number of proteins and lipids to label can also improve the study of endocytic and exocytic structures that often include multiple contributors. The use of a multi-labeling procedure such as DNA-based point accumulation for imaging in nanoscale topography (PAINT) that is suitable for super-resolution imaging can have vast potential in understanding relationships between more than four proteins or lipids in these phenomena [83–87]. The synergy of all the advanced light imaging techniques would benefit the spatiotemporal study of fusion and fission dynamics in a sophisticated manner.

## Perspectives

- The combination of various advanced light imaging techniques has led to many important scientific discoveries in the membrane dynamics of hemi and hemi-to-full fusion, fusion pore, fusing Ω-shape vesicular structure, and endocytic intermediate structural transitions in endocrine cells.
- These state-of-the-art light imaging techniques could be translated to the study of fusion and endocytosis well beyond endocrine cells, and the study of intracellular trafficking, viral fusion and endocytosis, as well as other membrane dynamics like cell migration and mitochondrial fission.
- Continued development of labeling techniques to minimize the effective size and imaging platforms with the higher spatiotemporal resolution are needed to facilitate the fundamental understanding of many biological processes.

## Abbreviations

A647: Alexa Fluor 647
CME: clathrin-mediated endocytosis
FFN: fluorescent false neurotransmitter
FRAP: fluorescence recovery after photobleaching
GFP: green fluorescent protein
MINFLUX: minimal photon fluxes
NPY: neuropeptide Y
NSF: *N*-ethylmaleimide-sensitive factor
PH: PHδ-phospholipase C domain
PIP2: phosphatidylinositol-4,5-bisphosphate
PM: plasma membrane
RRP: readily releasable pool
SNARE: soluble NSF attachment protein receptor
STAR: simultaneous two-wavelength axial ratiometry
STED: stimulated emission depletion
TIRF: total internal reflection fluorescence
VAMP2: vesicle-associated membrane protein 2
VMAT: vesicular monoamine transporter
